# Designing gene panels for tumor mutational burden estimation: the need to shift from ‘correlation’ to ‘accuracy’

**DOI:** 10.1186/s40425-019-0681-2

**Published:** 2019-08-06

**Authors:** Hao-Xiang Wu, Zi-Xian Wang, Qi Zhao, Feng Wang, Rui-Hua Xu

**Affiliations:** 1 Department of Medical Oncology, State Key Laboratory of Oncology in South China, Collaborative Innovation Center for Cancer Medicine, Sun Yat-Sen University Cancer Center, Guangzhou, 510060 People’s Republic of China; 2 Bioinformatics Platform, Department of Experimental Research, State Key Laboratory of Oncology in South China, Collaborative Innovation Center for Cancer Medicine, Sun Yat-Sen University Cancer Center, Guangzhou, 510060 People’s Republic of China

**Keywords:** Accuracy, Correlation, Panel, TCGA, Tumor mutational burden

## Abstract

Tumor mutational burden (TMB) assessment is at the forefront in precision medicine. The TMB could represent a biomarker for immune checkpoint inhibitors (ICIs) responses. Whole exome sequencing (WES) is the gold standard to derive the TMB; while targeted next-generation sequencing panels might be more feasible. However, mainstream panels use ‘correlation’ (R^2^) between panel- and WES-based TMB to validate TMB estimation, which could be vulnerable to be distorted by cases with relatively ultra-high TMB within each cancer type. The FDA-approved FoundationOne CDx (F1CDx) panel-based TMB estimation seemed reliable (R^2^ ≥ 0.75) in 24 out of 33 cancer types from the Cancer Genome Atlas, but most of them were overestimated by correlation as only seven cancer types had satisfactory accuracy (the proportion of cases correctly identified as TMB-high or TMB-low using panel-based TMB) above 90%. After removing cases with relatively ultra-high TMB within each cancer type, the correlation (R^2^) in 16 of these 24 cancer types declined dramatically (Δ > 0.25) while all of their accuracy remained generally constant, indicating that accuracy is more robust than correlation. Similar results were also observed in other four panels. Further incorporating accuracy in panel design revealed that the minimal number of genes needed to achieve ≥ 90% accuracy varied among cancer types and correlated negatively with their TMB levels (*p* = 0.001). In summary, currently available panels can accurately assess TMB only in several particular cancer types; and accuracy outperformed correlation in assessing the performance of panel-based TMB estimation. Accuracy and cancer type individualization should be incorporated in designing panels for TMB estimation.

**Electronic supplementary material:**

The online version of this article (10.1186/s40425-019-0681-2) contains supplementary material, which is available to authorized users.

## Background

The tumor mutational burden (TMB) is increasingly recognized as a potential biomarker for the response to immune checkpoint inhibitors (ICIs). Clinical studies have noted the association of high TMB with improved patient responses and survival benefit after ICI treatment either in a single cancer type (eg. non-small-cell lung cancer [1], melanoma [2], gastric cancer [3], and urothelial cancer [4]) or in combined cohort of multiple cancer types [5]. And the application of TMB as a biomarker for ICI treatment is now being prospectively tested [6, 7]. Therefore, TMB assessment has become a research hot spot in the field of precision medicine.

Currently, whole exome sequencing (WES)-derived TMB values are considered as the gold standard, but the high cost and long turnaround time limit the routine diagnostic applicability of WES. Therefore, targeted next-generation sequencing (NGS) panels have been promoted as a simpler and cheaper approach for TMB estimation [8]. Both the FDA-approved FoundationOne CDx (F1CDx) panel and the FDA-authorized MSK-IMPACT panel used ‘correlation’ (R^2^) between panel- and WES-based TMB to validate the capability of panel-based TMB estimation, and it’s claimed that these panels can assess TMB accurately (R^2^ = 0.74 for F1CDx and R^2^ = 0.76 for MSK-IMPACT) [9, 10]. Furthermore, Wang and colleagues recently claimed that a panel with more than 150 genes was sufficient for accurate TMB estimation based on their findings that the ‘correlation’ (R^2^) between the panel- and WES-based TMB gradually increased along with a decreasing standard deviation and reached a plateau after 150 genes were included [11]. However, the overall correlation between the panel- and WES-based TMB could be substantially distorted by outliers (i.e. cases with relatively ultra-high TMB within each cancer type) [12], which might lead to overestimation of the reliability of TMB estimation. Therefore, the present study aimed to assess the reliability of TMB estimation using these panels across multiple cancer types; and compared the robustness of ‘correlation’ and ‘accuracy’ in assessing the performance of panel-based TMB estimation.

## Methods

Ten thousand one hundred forty-seven cases across 33 cancer types from the Cancer Genome Atlas (TCGA) were included in this study. For WES mutation data, we used the uniform somatic called variants determined by TCGA MC3 project, which were comprehensively curated from detection using seven methods (MuTect, MuSE, VarScan2, Radia, Pindel, Somatic Sniper, Indelocator) [13]. The TMB was calculated as the number of non-synonymous somatic, coding, base substitution, and indel mutations per megabase (Mb) of genome examined, and 38 Mb was used as the estimate of the whole exome size [10]. Five currently available NGS panels for TMB determination (i.e. F1CDx, MSK-IMPACT, Illumina TSO500, Oncomine TML, QIAseq TMB) were investigated, and in silico simulated panel-based TMB scores were calculated by dividing the number of somatic mutations in the targeted genes by the region captured of corresponding panels per manufacturers’ instructions. Notably, for F1CDx and TSO500, synonymous mutations were also included in order to reduce sampling noise as the developers proposed. Previous studies have suggested that the inclusion of synonymous mutations could enhance the precision of panel-based TMB estimation [11, 14]. Still we preferred to retain the original algorithm for the other three panels without inclusion of synonymous mutations. We should also notice that although these panels have been developed for TMB determination, all of them, except for F1CDx and MSK-IMPACT, have not been approved by FDA as diagnostic assay and are still for research use only.

The primary outcomes were ‘correlation’ and ‘accuracy’. Correlations between panel- and WES-based TMB were examined using the Pearson correlation coefficient (R^2^). Accuracy was calculated as the proportion of cases that were correctly identified as either high TMB or low TMB using panel-based TMB. Besides accuracy, we additionally calculated the false positive rate (proportion of cases misclassified as TMB-high), false negative rate (proportion of cases misclassified as TMB-low), positive percentage agreement (calculated by dividing the number of true TMB-high by the total sum of true TMB-high and false TMB-low) and negative percentage agreement (calculated by dividing the number of true TMB-low by the sum of all true TMB-low and false TMB-high). Although retrospective analyses have established the predictive function of high TMB for a better response to ICIs, the optimal cutpoint to define high TMB varied among studies [15]. Based on the results of a multi-cancer cohort receiving ICI treatment, Samstein and colleagues proposed that there may not be one universal definition of high TMB; while the top 20% in each cancer type may serve as an option [5]. Thus, the top 20% in each cancer type was used as the cutpoint to define high TMB, and we varied the cutpoint from the top 10–50% for additional analysis.

As ‘correlation’ would be substantially distorted by cases with relatively ultra-high TMB (defined as cases with TMB ranking top 5% within a particular cancer type), we test the robustness of correlation and accuracy by successively removing cases with WES-based TMB ranking from the top 1–5% in each cancer type. In addition, we also examined the correlation between panel- and WES-based TMB in different TMB subgroups (top 5%, top 5–20%, and bottom 80%).

To explore the minimal number of genes needed for accurate TMB estimation (accuracy ≥ 90%) in each cancer type, we randomly extracted genes within the genomic scope to constitute randomized panels with size ranging from 150 to 1000 genes. The genes included in each size of panel were extracted randomly 1000 times. The minimal number of genes was truncated at 1000 for cancer types that needed more than 1000 genes to obtain a mean accuracy ≥ 90%.

## Results and discussion

**Fig. 1:**
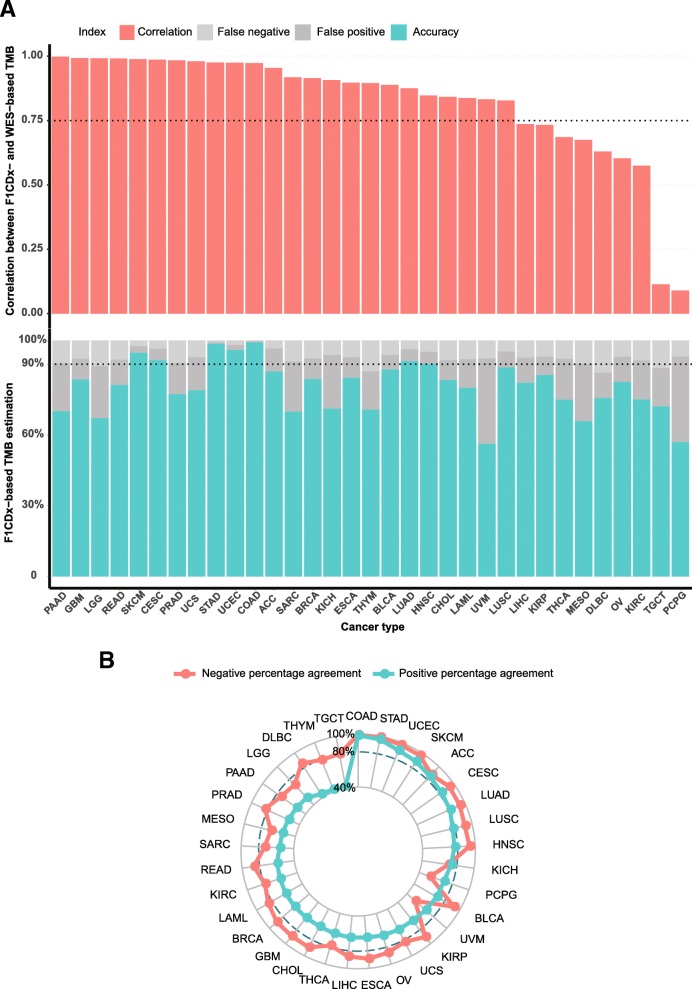
The reliability of F1CDx-based tumor mutational burden (TMB) estimation were overestimated by correlation. **a** The correlation between F1CDx- and WES-based TMB (top panel) and the accuracy, false positive rate, false negartive rate of F1CDx-based TMB estimation (bottom panel) across 33 cancer types in TCGA. **b** The positive percentage agreement and negative percentage agreement across 33 cancer types in TCGA

The correlations between the F1CDx- and WES-based TMB across 33 cancer types are shown in Fig. 1a, top panel. In accordance with previous studies [10], F1CDx seemed to accurately assess TMB (R^2^ ≥ 0.75) in at least 24 out of 33 cancer types. However, when using the top 20% in each cancer type as the cutpoint to define high TMB, the accuracy of these 24 cancer types ranged largely from 56 to 99% (Fig. 1a, bottom panel), and only seven cancer types had satisfactory accuracy (≥ 90%), while the false positive and false negative rate were considerable in other cancer types (Fig. 1a, bottom panel). Besides, the positive percentage agreement was below 80% in more than two-thirds of the 33 cancer types while the negative percentage agreements were rather high compared with corresponding positive percentage agreements (Fig. 1b). These results indicated that F1CDx-based TMB estimation was only reliable in particular cancer types (e.g. cervical squamous-cell carcinoma and endocervical adenocarcinoma [CESC], colon adenocarcinoma [COAD], head and neck squamous cell carcinoma [HNSC], lung adenocarcinoma [LUAD], skin cutaneous melanoma [SKCM], stomach adenocarcinoma [STAD], and uterine corpus endometrial carcinoma [UCEC]); while the reliability of F1CDx-based TMB estimation was overestimated by correlation in the other 17 cancer types with R^2^ ≥ 0.75. If we classified patients into TMB-high and TMB-low subgroups according to F1CDx-based TMB estimation in these 17 cancer types, considerable misclassification would happen, and mainly due to the misclassification of TMB-low patients as TMB-high (false positive).

The reason why the reliability of F1CDx-based TMB estimation was overestimated by correlation is that correlation is vulnerable to be distorted by the common presence of cases with relatively ultra-high TMB within each cancer type (Additional file 1: Figure S1). For example, there were 177 pancreatic adenocarcinoma (PAAD) cases in total, whose distribution of TMB (median [IQR], 0.92 [0.60–1.23] Mut/Mb) was presented in Additional file 1: Figure S1. Among these 177 PAAD cases, the F1CDx- and WES-based TMB estimation were highly correlated (R^2^ = 1.00). But if a relatively ultra-hypermutated case (TCGA-IB-7651) was omitted, the panel-based TMB estimation within the remaining 176 PAAD cases was found to be quite inaccurate and the correlation (R^2^) declined sharply to 0.33 (Additional file 1: Figure S2).

**Fig. 2:**
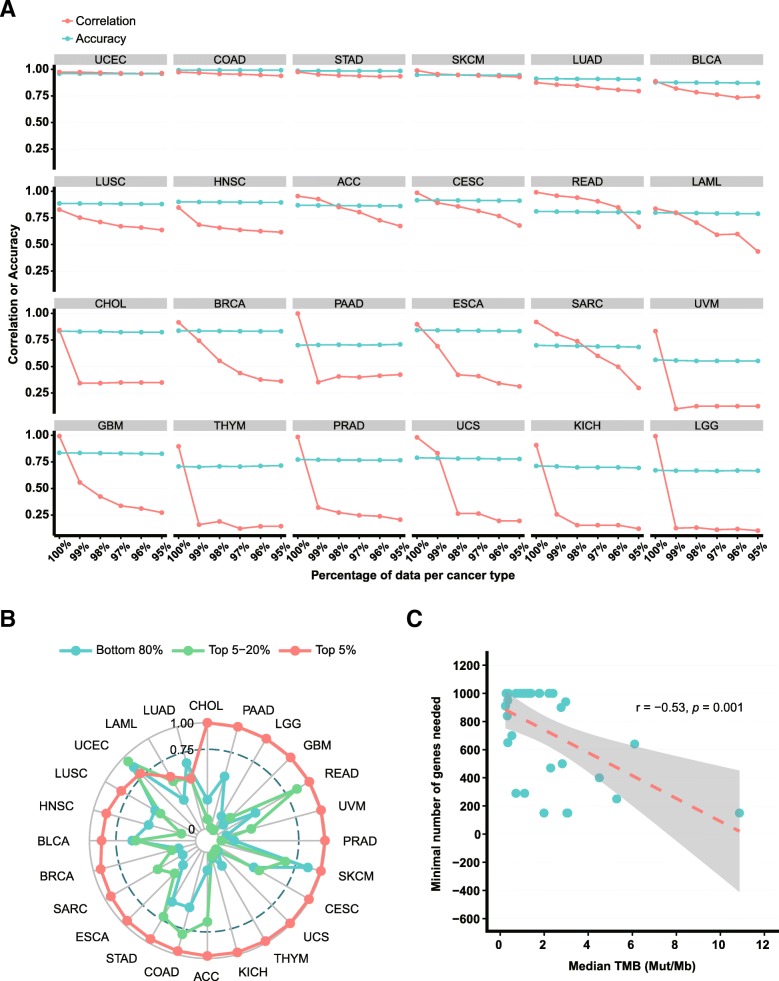
Accuracy outperformed correlation in assessing the performance of panel-based tumor mutational burden (TMB) estimation. **a** Changes in the correlation and accuracy when successively removing the cases with WES-based TMB ranking from the top 1–5% in each cancer type using the F1CDx panel. **b** The correlation between F1CDx- and WES-based TMB in different TMB subgroups (top 5%, top 5–20%, and bottom 80%) in 24 cancer types with R^2^ ≥ 0.75. **c** The minimal number of genes needed to obtain a mean accuracy ≥ 90% varied among cancer types and correlated negatively with their TMB levels

Therefore, we further tested the robustness of ‘correlation’ in assessing the performance of panel-based TMB estimation by removal of cases with relatively ultra-high TMB (defined as cases with TMB ranking top 5% within a particular cancer type). After successively removing the cases with WES-based TMB ranking from the top 1–5% in each of the 24 cancer types with R^2^ ≥ 0.75, a dramatic decline in correlation (Δ > 0.25) between F1CDx- and WES-based TMB was observed in more than half (16/24) of them (Fig. 2a).

In contrast, we successively removed the cases with WES-based TMB ranking from the top 1–5% in each cancer type, and found that the accuracy was generally constant in all 24 cancer types compared with correlation (Fig. 2a). Similar results were observed when we varied the cutpoint from the top 10–50% in each cancer type to define high TMB (Additional file 1: Figure S3). The superiority of accuracy over correlation was also prominent in other currently available NGS panels, e.g. MSK-IMPACT, Illumina TSO500, Oncomine TML, and QIAseq TMB panel (Additional file 1: Figure 4A-D). One may concern that the removal of top 5% cases systematically removed cases only from the TMB-high group and reduced the sample size of this group to 75%. Therefore, we also retained the top 5% cases and examined the correlation between F1CDx- and WES-based TMB in different TMB subgroups (top 5%, top 5–20%, and bottom 80%). As shown in Fig. 2b, in more than two-thirds of 24 cancer types with R^2^ ≥ 0.75, the correlations between F1CDx- and WES-based TMB estimation in the top 5–20% subgroup and the bottom 80% subgroup were similar, but both were lower than that in the top 5% subgroup and the total cases, indicating that the correlation was distorted by cases with relatively ultra-high TMB and the reliability of panel-based TMB estimation was actually suboptimal in these cancer types. Additional analysis using the other four NGS panels confirmed this result (Additional file 1: Figure 5A-D).

These results strongly suggested that accuracy was a robust and better index compared with correlation in assessing the performance of panel-based TMB estimation and could be readily incorporated into the design of panels for TMB estimation.

In previous studies, the performance of panel-based TMB estimation were examined within limited sample size (*n* = 29 for F1CDx and *n* = 106 for MSK-IMPACT) [9, 10]. Based on the in silico analysis of well-curated WES data from more than 10,000 cases, we found that the precision of F1CDx or other panels-based TMB estimation might not be adequate in all cancer types, especially in those with intermediate to low TMB levels (Additional file 1: Figure S6), in most of which the reliability of panel-based TMB was otherwise overestimated by ‘correlation’. Therefore we should not validate panels for TMB estimation simply relying on ‘correlation’ as sometimes it could give rise to misleading results, which would probably cause improper application of ICIs. Besides, the accuracy of F1CDx-based TMB estimation varied among cancer types and positively correlated with their TMB levels (Additional file 1: Figure S6, *p* < 0.001), indicating that one universal NGS panel may not be enough for TMB estimation across multiple cancer types, while cancer type individualized panels accounting for their TMB levels could be more applicable.

‘Correlation’ is a measure of the linear relationship between two variables and can be readily interpreted. For assessment of panels, the higher the correlation is, the more precise the panel would be. But it is often distorted by cases with relatively ultra-high TMB, which cannot be avoided in most cancer types. While ‘accuracy’ is based on the method of dichotomy, thus it would not be significantly affected by outliers, and is more applicable in clinical settings. Besides the precise estimation of TMB values, we would focus more on how many patients will be incorrectly classified as TMB-high (false positive) and TMB-low (false negative). An accuracy of 90% (e.g. HNSC) means that 10% of the patients would be misclassified according to the results of F1CDx and consequently leads to improper decisions on the application of ICIs in these patients. Meanwhile, as a method of dichotomy, ‘accuracy’ focuses more on the proportion of misclassification rather than the exact TMB estimation of every sample; and the cutopoint needs to be prespecified. Although the continuum of TMB values also matters as the survival benefit was more pronounced when TMB cutpoint got higher [5], definite cutpoints for TMB may be more practical and interpretable in clinical settings. Another effective biomarker for ICI treatment, PD-L1 expression, also obtained approval based on definite cutpoints. Thus the better option might be carefully combining ‘accuracy’ along with ‘correlation’ in the validation of NGS panels.

To give an example of incorporating accuracy into the design of panels for TMB estimation, we randomly extracted genes to generate virtual panels of 150 to 1000 genes to explore the minimal number of genes needed for accurate TMB estimation. In most cancer types, the mean accuracy gradually increased but few reached a plateau (Additional file 1: Figure S7). The minimal number of genes needed to obtain a mean accuracy above 90% varied among cancer types (median [range], 940 [150–1000]) and correlated negatively with their TMB levels (Fig. 2c, *p* = 0.001), which was in line with our proposal that panels for TMB estimation should be cancer type individualized in terms of cost and benefit. For cancer types with higher TMB levels, smaller panels are sufficient to capture the mutational burden, while for cancer types with lower TMB levels, larger panels are needed. Certainly, randomly selected gene panels may not be appropriate for TMB estimation, and it’s not cost-effective to develop a NGS panel only for TMB estimation. As mutational spectrum is divergent across cancer types, cancer type individualized panels in which the size (how many genes) and composition (what genes) are carefully elaborated would be more applicable both for TMB estimation and identification of actionable targets.

It’s thought-provoking that the first prospective clinical trial (CheckMate 227) seems to fail in establishing the predictive function of TMB, probably due to the dilution of treatment effect caused by misclassification of TMB-high and TMB-low patients by F1CDx-based TMB estimation. The key usage of ‘accuracy’ is that it is robust in assessing the reliablity of panel-based TMB albeit the common presence of outliers. As using NGS panels to determine TMB is more feasible than WES, a panel with high accuracy could reduce the misclassification in clinical trials, thus guarantees greater power in detecting the predictive function of TMB and establishes validated TMB cutpoints.

A major limitation of this study is that there are still lots of pre-analytic issues about the clinical application of panel-based TMB. For instance, the variation of sample storage time, the high scoring failure rate, and so on [16]. Therefore, incorporating the methods of ‘accuracy’ and ‘cancer type individualization’ in panel design requires wet-lab validation before it could be used in clinical practice.

## Conclusions

Increasing numbers of clinical trials include the TMB as a key design component; therefore, accurate TMB assessment is fundamental to ensure reliable and reproducible identification of those patients likely to benefit from ICI treatment. The present study showed that, the currently available NGS panels can assess TMB accurately only in several particular cancer types; and with the presence of cases with relatively ultra-high TMB, ‘correlation’ is unreliable to evaluate the performance of panel-based TMB estimation in most cancer types, whereas ‘accuracy’ is a superior index in this situation. Furthermore, cancer type individualized panels might be a better strategy to guarantee robust TMB estimation and thus greater power in prospectively detecting the predictive function of TMB across multiple cancer types.

## Additional file

Additional file 1: **Figure S1.** The tumor mutational burden across 33 cancer types in TCGA. **Figure S2.** The correlation between F1CDx- and WES-based TMB in PAAD after the removal of a relatively ultra-hypermutated case. **Figure S3.** Changes in the accuracy with cutpoint varying from the top 10–50% when successively removing the cases with WES-based TMB ranking from the top 1–5% in each cancer type. **Figure S4.** Changes in the correlation and accuracy when successively removing the cases with WES-based TMB ranking from the top 1–5% in each cancer type using the MSK-IMPACT panel (A), the Illumina TSO500 panel (B), the Oncomine TML panel (C), and the QIAseq TMB panel (D). **Figure S5.** The correlation between panel- and WES-based TMB in different TMB subgroups (top 5%, top 5–20%, bottom 80%), the MSK-IMPACT panel (A), the Illumina TSO500 panel (B), the Oncomine TML panel (C), and the QIAseq TMB panel (D). **Figure S6.** The accuracy of F1CDx-based TMB estimation varied among cancer types and correlated positively with their TMB levels. **Figure S7.** The mean accuracy of panels comprising 150 to 1000 genes gradually increased but few reached a plateau. (PDF 156 kb)

## Data Availability

The TCGA MC3 Public MAF is available at https://gdc.cancer.gov/about-data/publications/mc3-2017.

## Abbreviations

ACC: Adrenocortical carcinoma
BLCA: Bladder urothelial carcinoma
BRCA: Breast invasive carcinoma
CESC: Cervical squamous-cell carcinoma and endocervical adenocarcinoma
CHOL: Cholangiocarcinoma
COAD: Colon adenocarcinoma
DLBC: Lymphoid neoplasm diffuse large b-cell lymphoma
ESCA: Esophageal carcinoma
F1CDx: FoundationOne CDx
GBM: Glioblastoma multiforme
HNSC: Head and neck squamous cell carcinoma
ICIs: Immune checkpoint inhibitors
KICH: Kidney chromophobe
KIRC: Kidney renal clear cell carcinoma
KIRP: Kidney renal papillary cell carcinoma
LAML: Acute myeloid leukemia
LGG: Brain lower grade glioma
LIHC: Liver hepatocellular carcinoma
LUAD: Lung adenocarcinoma
LUSC: Lung squamous cell carcinoma
Mb: Megabase
MESO: Mesothelioma
NGS: Next-generation sequencing
OV: Ovarian serous cystadenocarcinoma
PAAD: Pancreatic adenocarcinoma
PCPG: Pheochromocytoma and paraganglioma
PRAD: Prostate adenocarcinoma
READ: Rectum adenocarcinoma
SARC: Sarcoma
SKCM: Skin cutaneous melanoma
STAD: Stomach adenocarcinoma
TCGA: The Cancer Genome Atlas
TGCT: Testicular germ cell tumors
THCA: Thyroid carcinoma
THYM: Thymoma
TMB: Tumor mutational burden
UCEC: Uterine corpus endometrial carcinoma
UCS: Uterine carcinosarcoma
UVM: Uveal melanoma
WES: Whole exome sequencing
