# Supplementary material for: Designing gene panels for tumor mutational burden estimation: the need to shift from ‘correlation’ to ‘accuracy’
Source: J Immunother Cancer. 2019 Aug 6;7:206. doi: 10.1186/s40425-019-0681-2 (PMC6685228; doi:10.1186/s40425-019-0681-2)

Figure S1. The tumor mutational burden across 33 cancer types in TCGA.

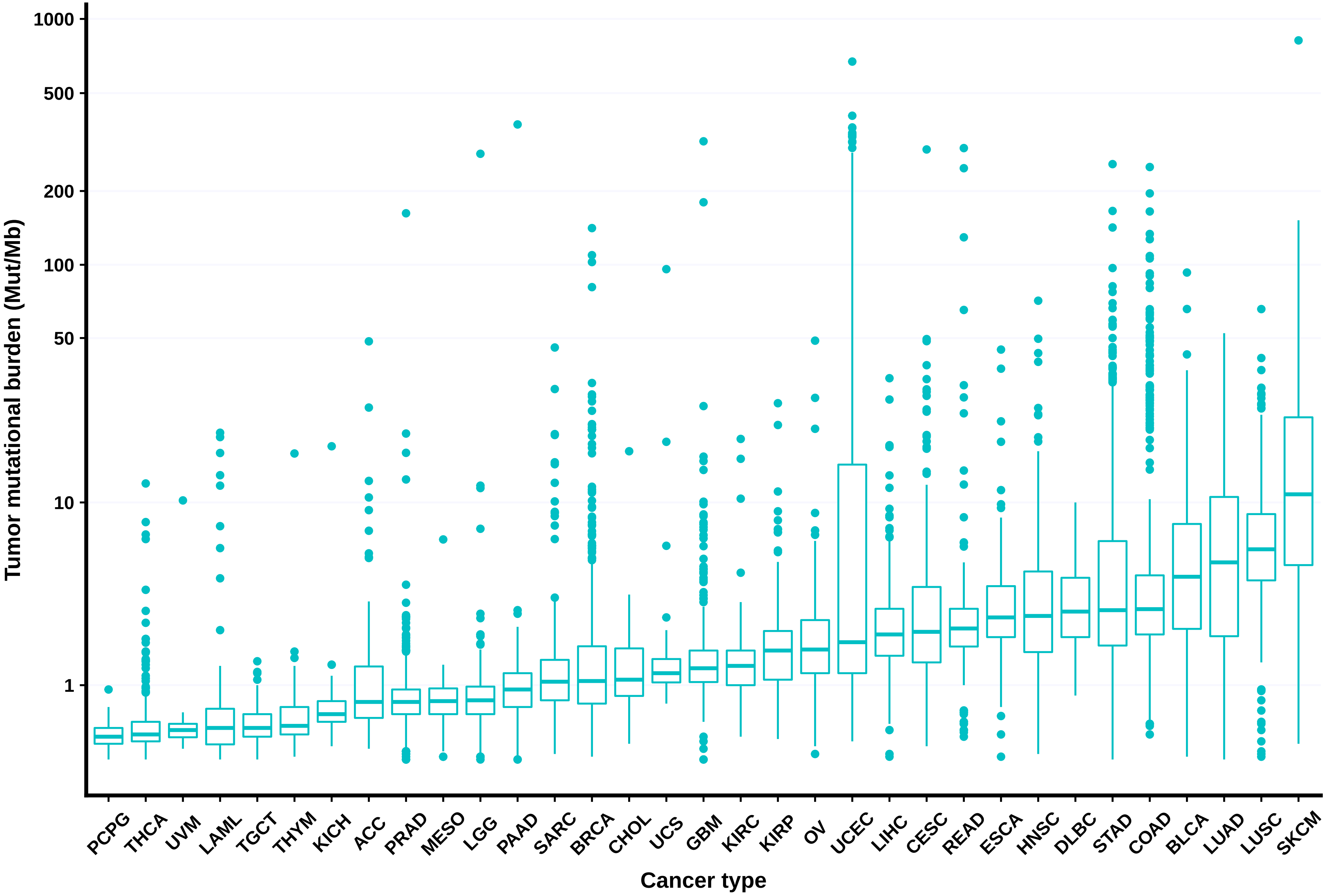

Figure S2. The correlation between F1CDx− and WES−based TMB in PAAD after the removal of a relatively ultra−hypermutated case.

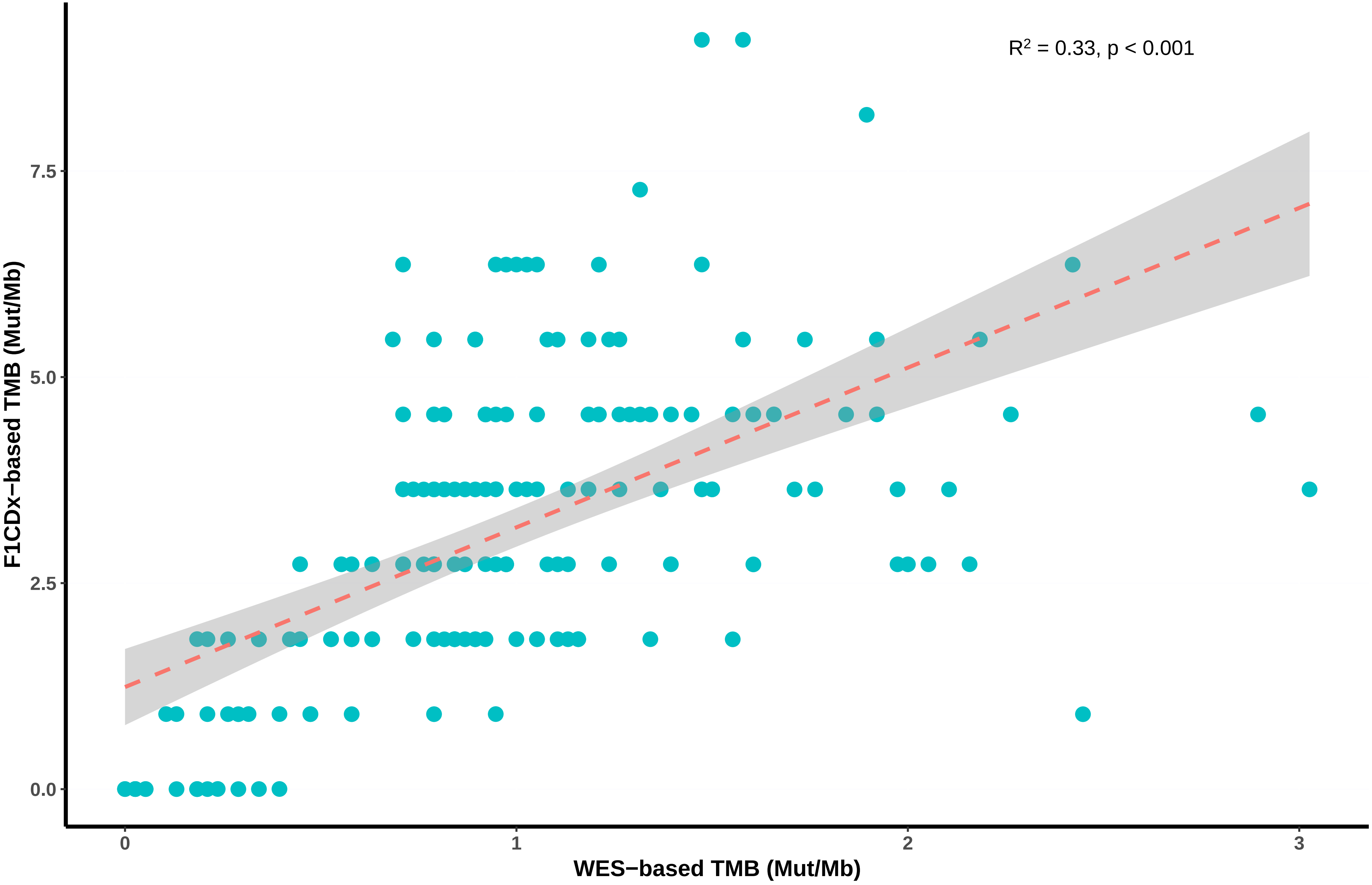

Figure S3. Changes in accuracy with the cutpoint varying from the top 10−50% when successively removing the cases with WES−based TMB ranking from the top 1−5% in each cancer type.

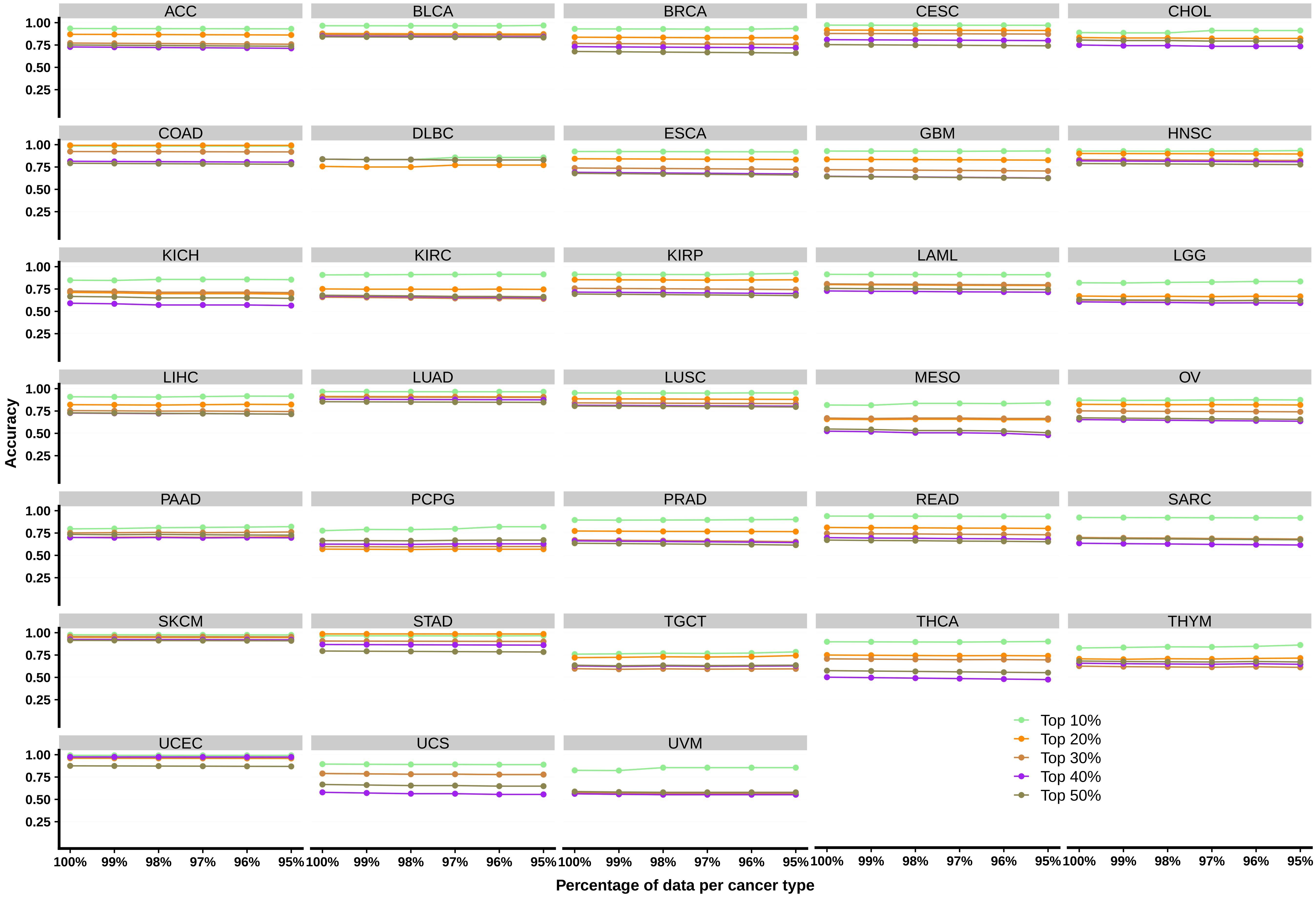

Figure S4A. Changes of correlation and accuracy when successively removing the cases with WES−based TMB ranking from top 1−5% in each cancer type using MSK−IMPACT panel.

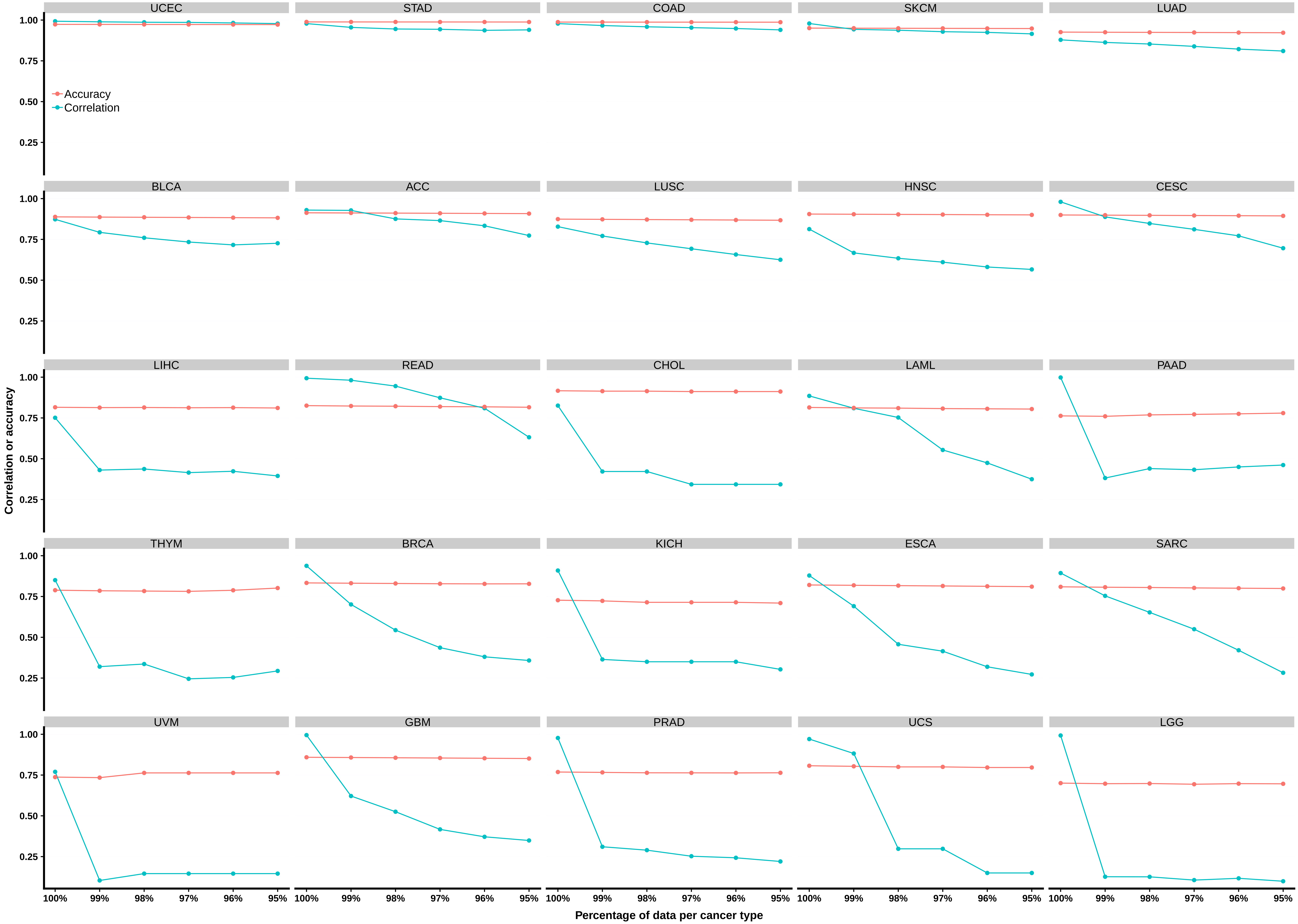

Figure S4B. Changes of correlation and accuracy when successively removing the cases with WES−based TMB ranking from top 1−5% in each cancer type using TSO500 panel.

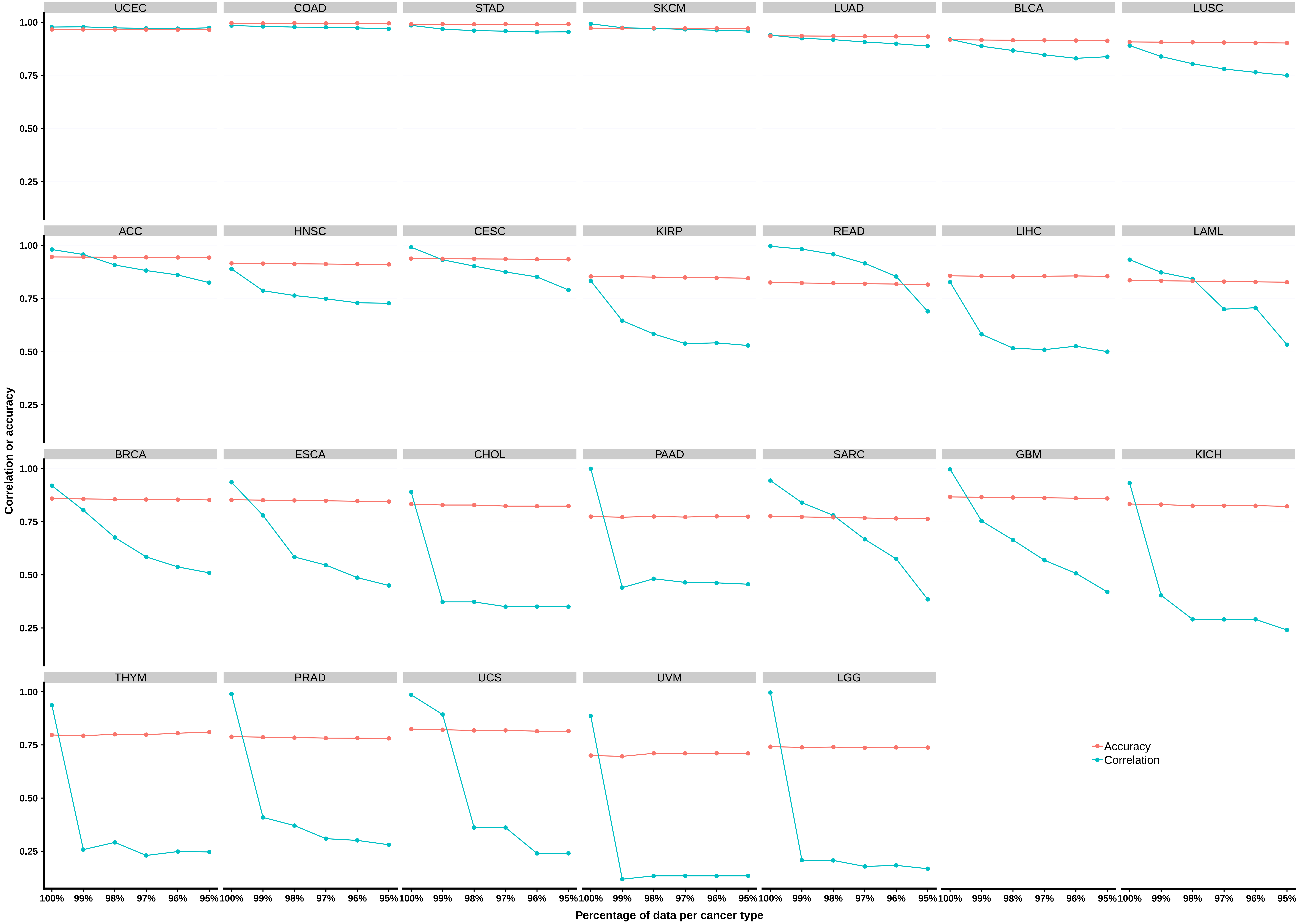

Figure S4C. Changes of correlation and accuracy when successively removing the cases with WES−based TMB ranking from top 1−5% in each cancer type using Oncomine TML panel.

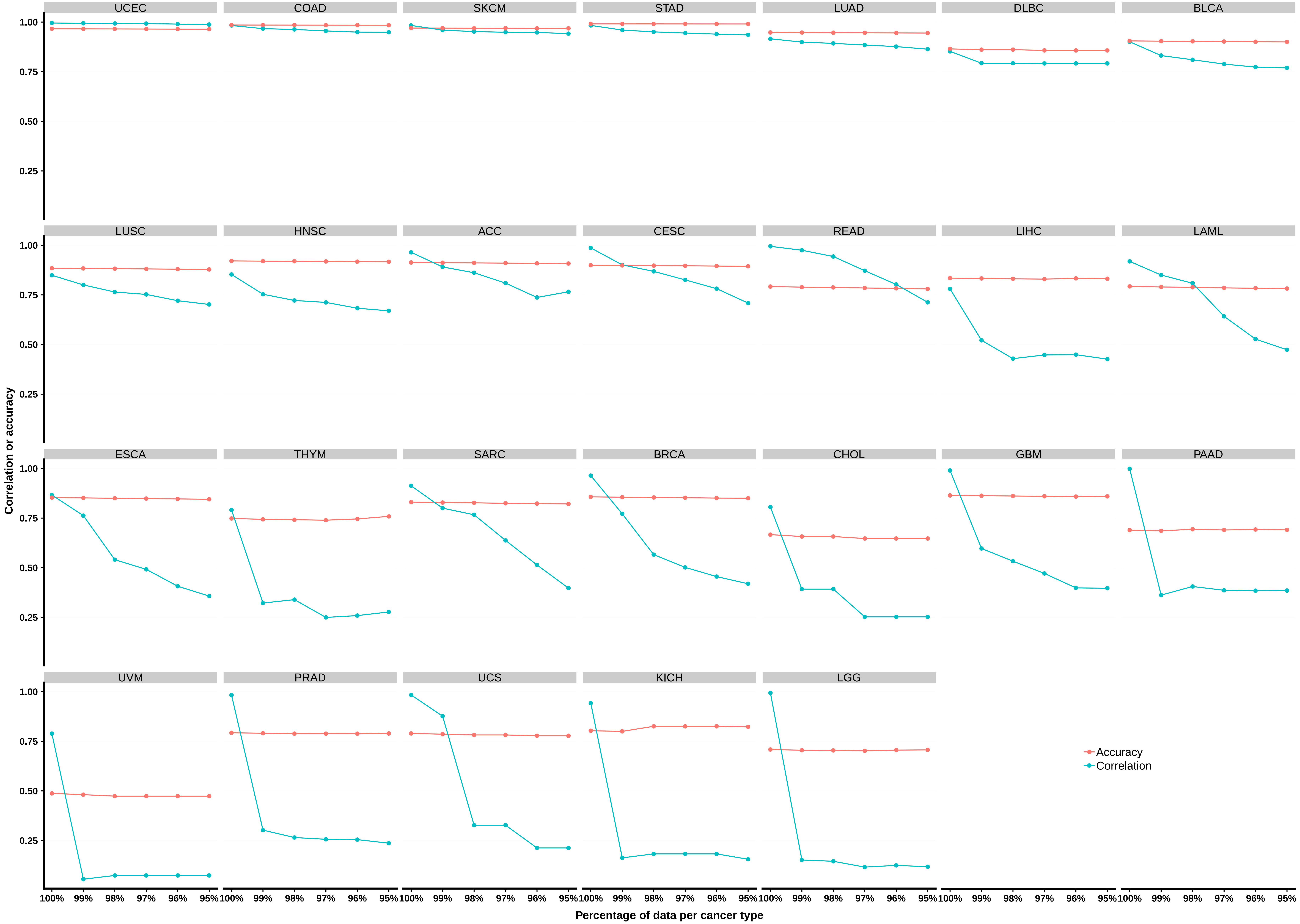

Figure S4D. Changes of correlation and accuracy when successively removing the cases with WES−based TMB ranking from top 1−5% in each cancer type using QIAseq TMB panel.

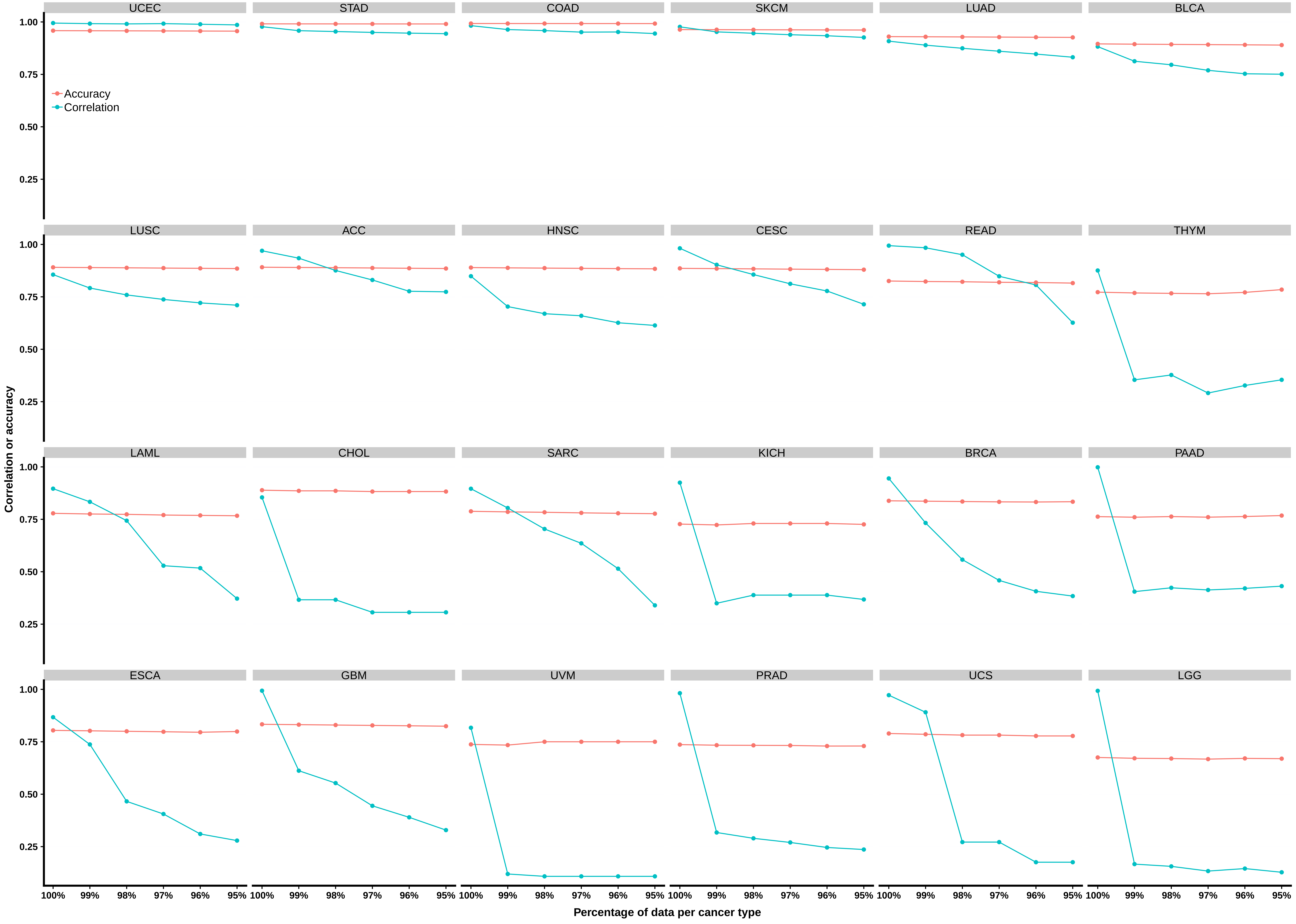

Figure S5A. Correlation between MSK−IMPACT− and WES−based TMB estimation in different TMB subgroups.

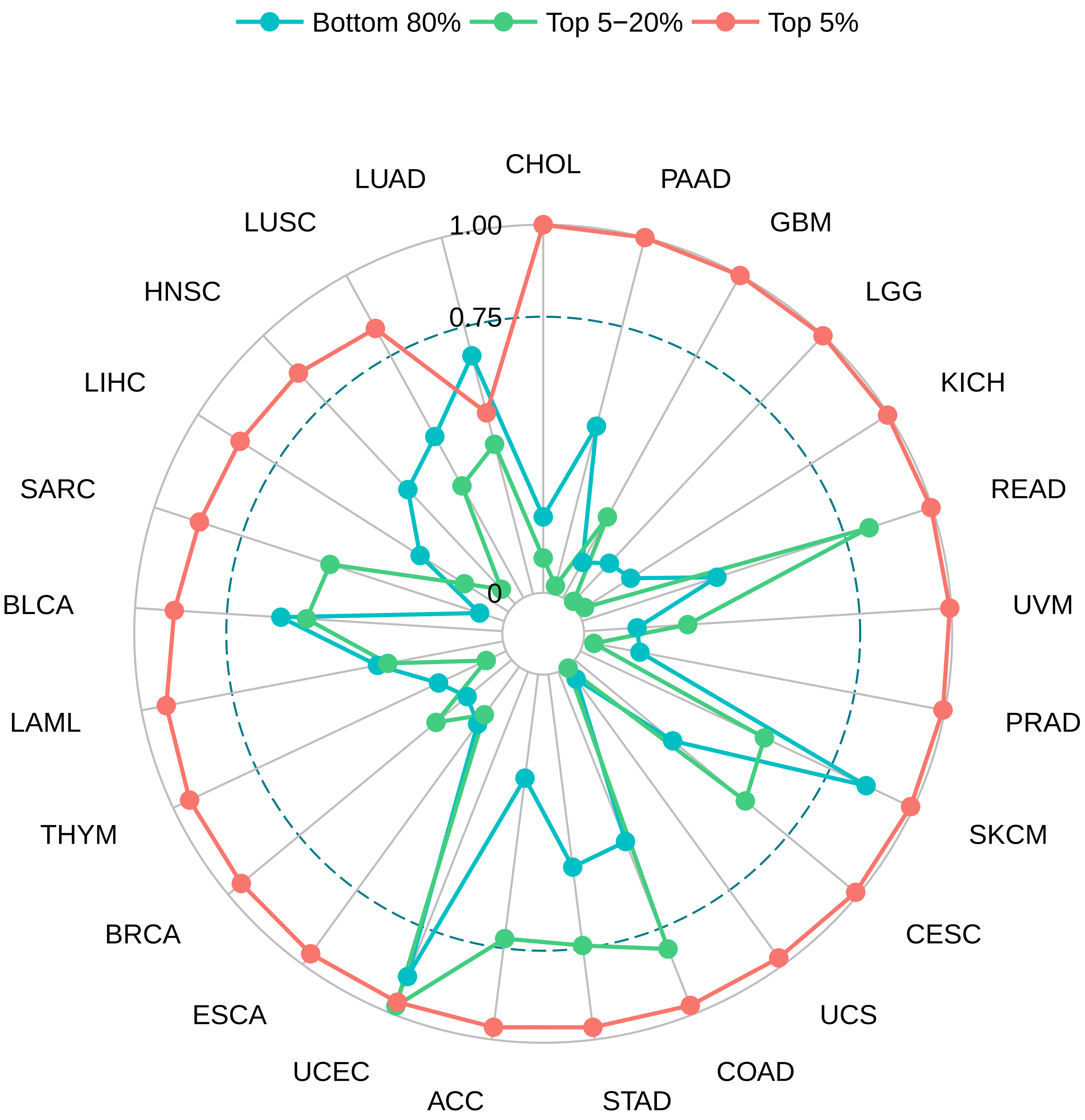

Figure S5B. Correlation between TSO500− and WES−based TMB estimation in different TMB subgroups.

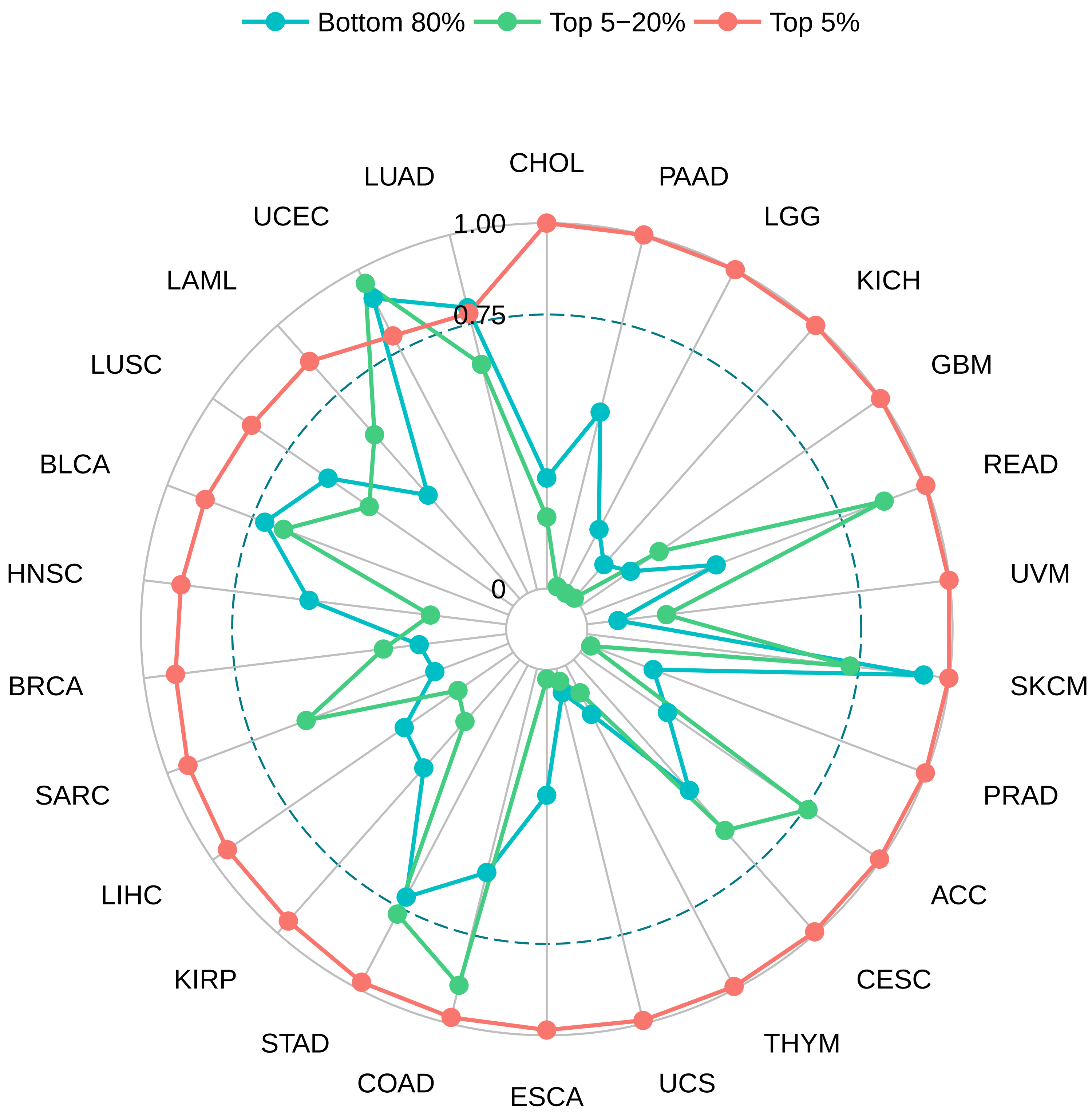

Figure S5C. Correlation between Oncomine TML− and WES−based TMB estimation in different TMB subgroups.

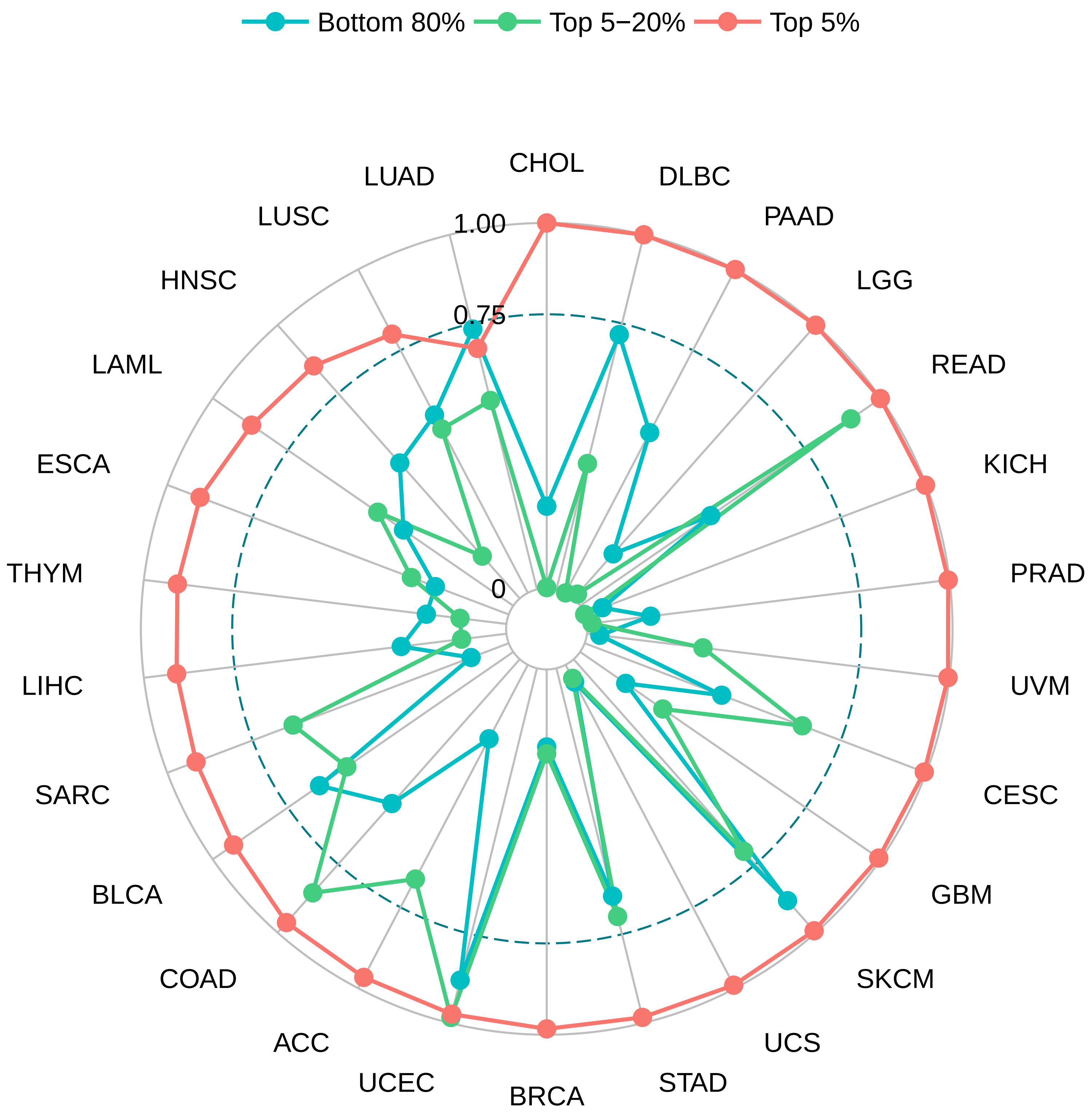

Figure S5D. Correlation between QIAseq TMB− and WES−based TMB estimation in different TMB subgroups.

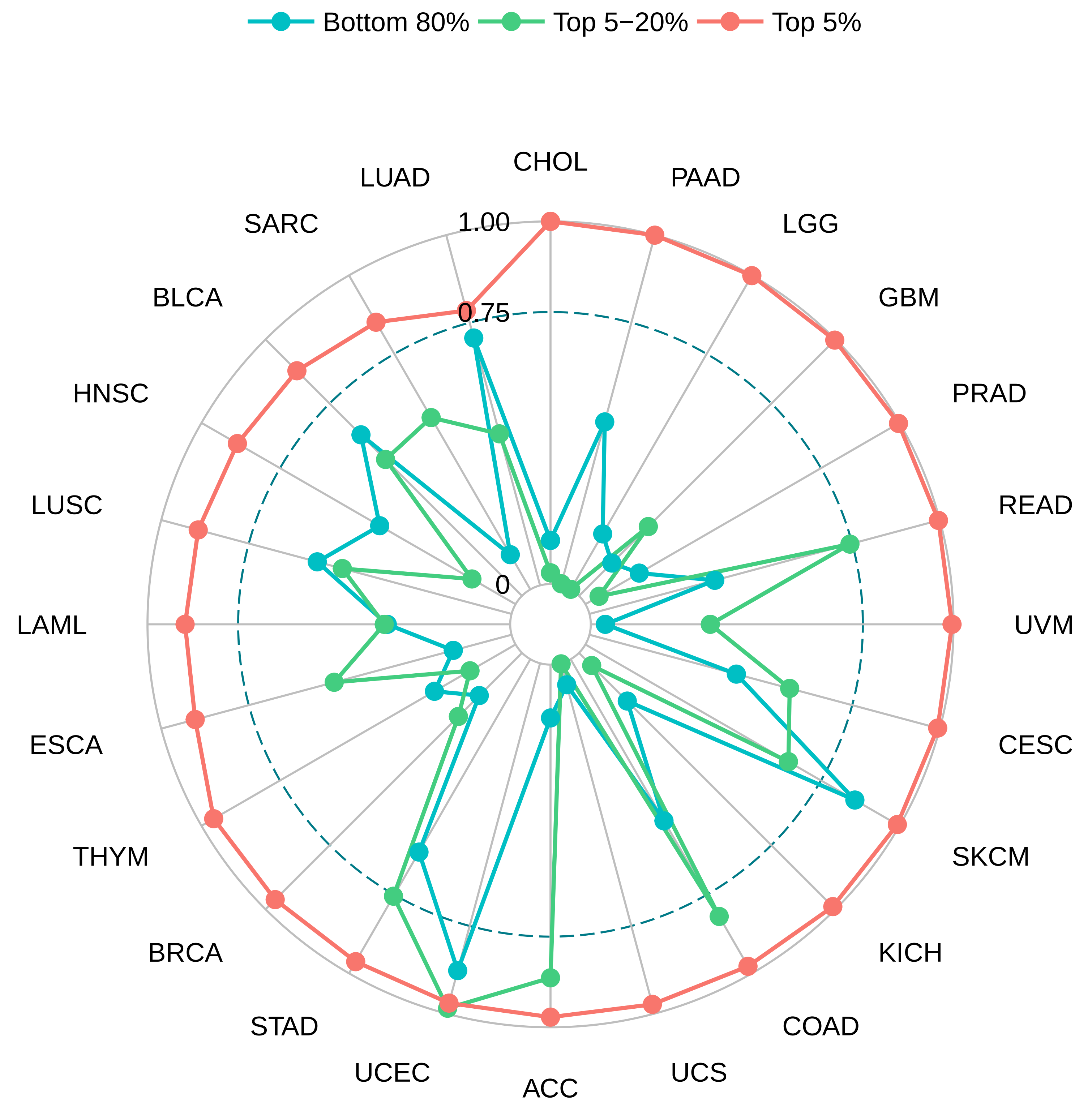

Figure S6. The accuracy of F1CDx-based TMB estimation varied among cancer types and correlated positively with their TMB levels.

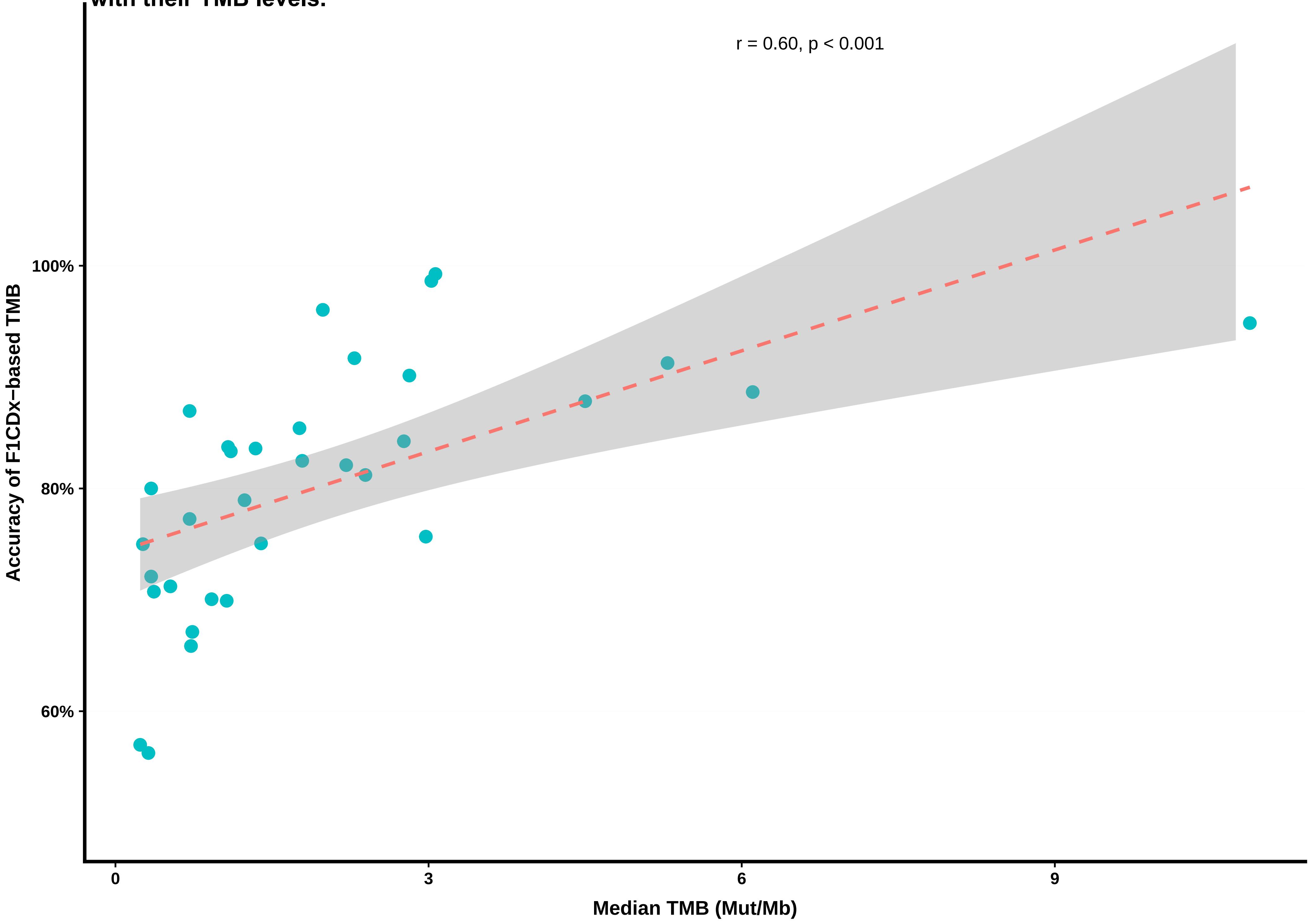

Figure S7. The mean accuracy of panels with size from 150 to 1000 genes gradually increased but few reached plateu.

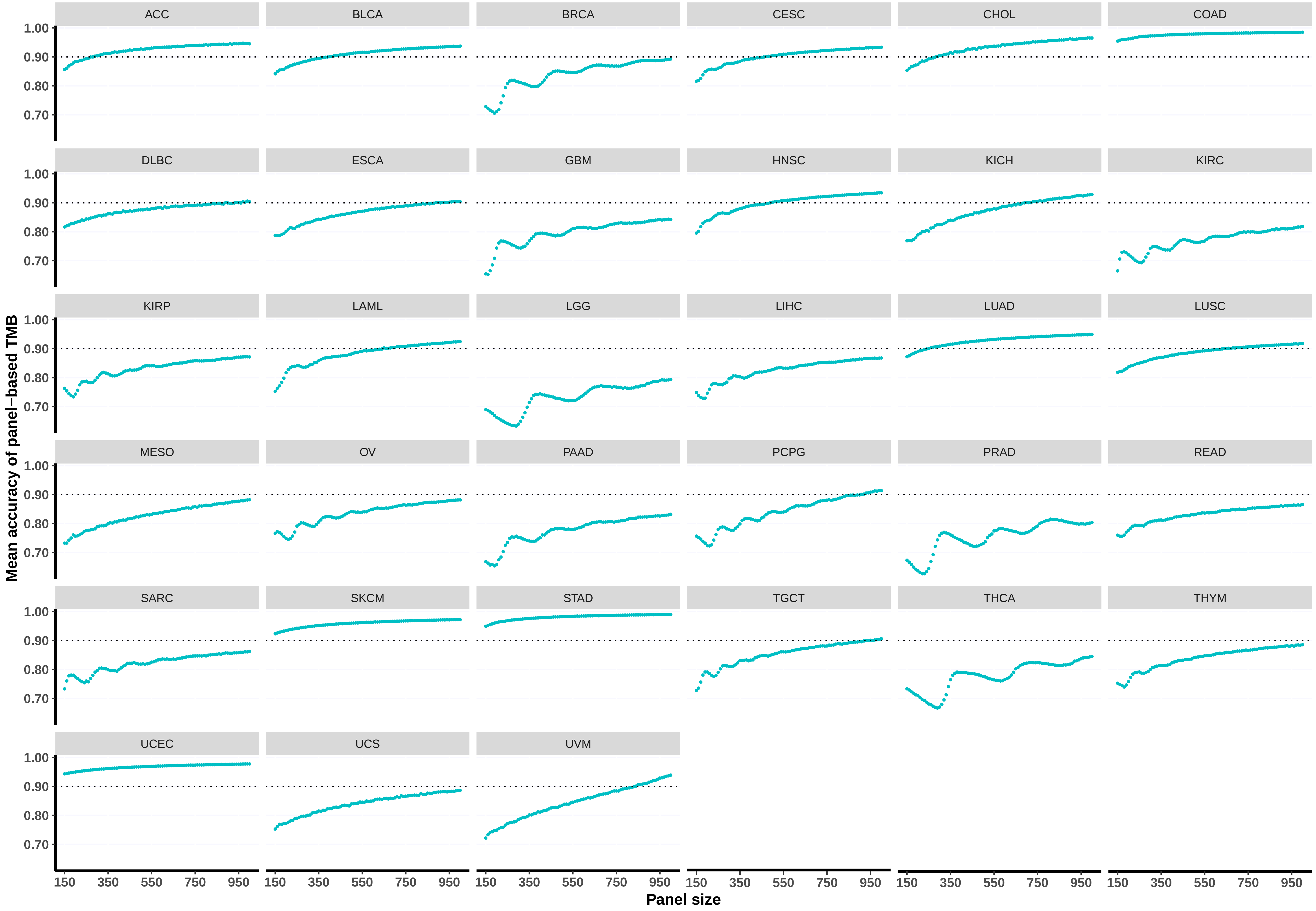

Supplement: Supplementary file 1 — Figure S1. The tumor mutational burden across 33 cancer types in TCGA. Figure S2. The correlation between F1CDx- and WES-based TMB in PAAD after the removal of a relatively ultra-hypermutated case. Figure S3. Changes in the accuracy with cutpoint varying from the top 10–50% when successively removing the cases with WES-based TMB ranking from the top 1–5% in each cancer type. Figure S4. Changes in the correlation and accuracy when successively removing the cases with WES-based TMB ranking from the top 1–5% in each cancer type using the MSK-IMPACT panel (A), the Illumina TSO500 panel (B), the Oncomine TML panel (C), and the QIAseq TMB panel (D). Figure S5. The correlation between panel- and WES-based TMB in different TMB subgroups (top 5%, top 5–20%, bottom 80%), the MSK-IMPACT panel (A), the Illumina TSO500 panel (B), the Oncomine TML panel (C), and the QIAseq TMB panel (D). Figure S6. The accuracy of F1CDx-based TMB estimation varied among cancer types and correlated positively with their TMB levels. Figure S7. The mean accuracy of panels comprising 150 to 1000 genes gradually increased but few reached a plateau. (PDF 156 kb) [file 40425_2019_681_MOESM1_ESM.pdf]
